# Surveys about attended births appear to be deceptive in CAR: are the population saying what they think NGO’s want to hear?

**DOI:** 10.1186/s13031-021-00381-6

**Published:** 2021-06-13

**Authors:** Philippe Wol, Christina Kay, Leslie Roberts

**Affiliations:** 1 Independent Consultant, Bocaranga, Central African Republic; 2 Program on Forced Migration and Health, Columbia University – Mailman School of Public Health, New York, USA

## Abstract

**Background:**

Non-governmental organizations (NGOs) and donors often promote certain practices to a community, such as in-facility births and then evaluate the efficacy of those interventions, in part, by surveying those populations.

**Methods:**

A project to assess the accuracy of birth and death monitoring by local community-based monitors was undertaken with a partner health agency in areas (pop. 94,000) where they supported medical facilities. Thirty clusters of 30 households each were selected at random, probability proportional to size. Half of those households were enrolled for a monthly visitation surveillance process. To gain insights into the effects of the agency’s services, an additional 240 households were selected at random and interviewed from 8 nearby “matched villages” not serviced by any NGO as a comparison sample.

**Results:**

The 896 households with 4243 living residents within the NGO service area were interviewed about household births and deaths within the past 8 months. They reported an annualized birth rate of 5.6% (95% CI: 4.5–6.7) with only 3% of those births occurring at home. The reported death rate was 4.2/1000/month (95% CI: 3.3–5.0). In the “matched villages,” the population reported a similar birth and death rate, but they reported 29% of births occurring within the home. The monthly surveillance data found over the year that followed that 32% of births occurred at the home. Clinic and hospital birth attendance data suggested an attended annual birth rate of only 2.8%, consistent with the surveillance data implication that a huge fraction of births occur at home.

**Conclusion:**

It is believed that because the baseline interviews occurred with a stranger, this induced interviewees to say what they thought the interviewers wanted to hear. This calls into question the validity of household surveys when agencies have a known agenda or position, and highlights the need for external validation or triangulation of survey findings.

## Background

There is a long history of refugees and conflicted affected people needing to be deceptive or misleading in order to survive [1]. In some cases, the deception arises because aid is targeted towards specific groups, like child soldiers or survivors of sexual assault, and other people in desperate need become incentivized to categorize themselves as such [2]. For example, a report about Eastern DRC described women falsely reporting that they had been raped to receive relief aid, and then sharing that aid with women who actually had been raped but for culture and stigma reasons felt unable to disclose this to NGO workers [3]. Such observations are rarely reported by humanitarian agencies for a variety of reasons including loss of donor support.

The Central African Republic is one of the world’s poorest countries [4]. Approximately 70% of the country’s area has been outside of the Government’s control for more than a decade and multiple measures have found very high mortality estimates in these rebel held areas [5–7]. Surveillance systems in these rebel held areas are generally believed to be insensitive. For example, the UN’s Monitoring and Reporting Mechanism (MRM) for detecting the murder, rape and abduction of children by armed groups was found to be less than 1% complete in 2010 [8]. In these areas, most health services are provided by non-governmental organizations.

A project between Columbia University’s Program on Forced Migration and Health and the International Rescue Committee (IRC) was undertaken to establish a surveillance system to record births, deaths and migration in those parts of Ouham-Pende Prefecture served by IRC’s health programs. Community-based surveillance has shown in this region to provide higher quality birth and death data than traditional surveys [9]. At the time this data was collected, IRC had been in Ouham-Pende for a decade and provided a variety of services, including clinic support. IRC provided drugs, equipment, supervision, and support for community outreach to 11 health facilities. IRC covered the costs of all basic health care services for children less than 5 years of age, and for all pregnancy and birth-related health care. In several communities, IRC also built and funded new maternal care facilities to allow for facility-based deliveries within these rural towns. The aim of this study was to compare findings from the baseline survey in August–September 2018 and the data from household surveillance monitoring conducted over the year that followed.

## Methods

Data were collected from three procedures for this study: household surveys, monthly monitoring, and traditional administrative surveillance. A baseline cluster survey was conducted that represented the IRC service areas, and in 8 other locations, that were matched control villages not served by IRC. Then, a subset of those households in the IRC service areas were visited on a monthly basis, hereafter called surveillance households. Finally, data had been and was collected by IRC, the Ministry of Health, and other service organization as part of their ongoing programs, hereafter referred to as “administrative data.”

In the baseline survey, thirty sample clusters were assigned probability proportional to size for monitoring to the 14 clinic populations (total pop = 94,000) served by IRC in Ouham-Pende Prefecture in August of 2018. These hospitals and health-posts are in the Sous Prefectures of Bocaranga, Ngaoundaye, and Koui. Seventeen of the 30 clusters were in or near the three large towns of Bocaranga (pop. 16,000), Ngaoundaye (pop. 19,000), and Koui (pop. 17,000), which each had a hospital with inpatient facilities, while the other 13 clusters were in eight towns with a median population of 4000 and only an outpatient health-post or center. Five of the eight smaller towns also had IRC maternal care facilities which could perform cesarean sections and provided maternity-related inpatient care.

The sample size estimate assumed there would be four people per household, that 2% of the population gave birth attended by an IRC supported professional each year, a design effect of 2, and that this could document the birth rate with a precision of 0.5% with 95% confidence and 80% power. It was determined that a larger sample was needed for the baseline survey than would be needed for the surveillance project, which was expected to run for 2 years.

Within each village, a coin toss was used to subdivide the village down to an area with > 30 households. Usually, the subdivision began in the center of the village, often with a second subdivision. From the selected point, the first 15 households were selected in a North-easterly direction (that is, within a 90 degree angle between North and East) to be interviewed and enrolled in the monthly surveillance process and consent was solicited and obtained. These will be referred to as surveillance households and were roughly half of the households that constituted the baseline survey.

In order to obtain the adequate sample size desired in the baseline survey, an additional occupied 15 households were interviewed, closest to the starting point but in the quadrant between North and West, and consent just for the interview process was solicited and obtained from them. Because villages were almost never more than 20 households across, this process usually spanned from a central road or path to the edge of the village. If the interviewer visited all households within their quadrant and did not reach 15 households (rare), they returned to the original point and expanded their quadrant to the South until 15 interviews were completed. In most cases, after completing 10 or 12 of the 15 interviews, the interviewers would return to the empty houses in case some occupants had returned to minimize the fraction that was not interviewed. An attempt was made to record the fraction of homes that were empty vs. abandoned. Including the initial meetings with the village chiefs, each 30 household cluster took about 5 h to complete using four interviewers.

As part of the baseline survey, households were asked to list the age and sex of all household members and if anyone had given birth or died since last Christmas (a recall of 7.7 months). If births or deaths had occurred, a series of follow-on questions recorded if they had attended a clinic or hospital and what the symptoms or cause of illness had been. No formal verbal autopsy process was followed and no confirmation of the household report was sought.

As a secondary element of the baseline survey, eight additional villages not served by IRC, on the same axis, but more than 10 km away from an IRC clinic were also visited. These “matched control” villages tended to not have a health facility, but in three cases had a facility run by the Ministry of health and not supported by an NGO beyond drug provision. The same baseline survey procedure was employed, and 30 households were selected and interviewed. No ongoing surveillance was established in these communities. This “matched control village” sampling was done to be able to compare attended birth rates and death rates in the IRC served populations with villages not served by any NGO.

For the households enrolled in the monthly surveillance process that occurred over the year-and-a-half that followed, they were re-visited each month by the same monitor. In the three largest towns (17 clusters), this was conducted by two project employees who had been interviewers in the baseline survey. In most of the smaller villages, a village resident, selected by the village chief was the local monitor. Local monitors were initially trained by the project manager for approximately 1 h and then accompanied the team to be introduced to the households and observe the baseline interviews. Before the first round of monthly surveillance, an all-day training was conducted in the Prefecture capital. Finally, during the first round of monthly visits, the project manager accompanied, observed, and coached the local monitor regarding the interviewing and information recording. Monthly visits inquired about any pregnancies, births, deaths, or migration into or out of the household since the last visit the month before. Usually these visits happened just after sunrise or in the hour before sunset and over time the monitors evolved to know which households would be home when. Three or four return visits to a cluster over the course of a couple of days was common, allowing for much lower absenteeism rates compared with the baseline survey. When entire households moved away, the nearest house not under surveillance was recruited for inclusion in the surveillance study, although this often took a couple months. The local monitors were visited once a month by the project manager who recorded their monthly data and discussed any issues or complicated cases. While some variation occurred over time, there have been 11 monthly monitors in total, two of whom had been baseline interviewers.

Administrative data regarding births attended was obtained from monthly reports provided by clinics and hospitals to IRC. Estimates of unattended births came from IRC’s network of health outreach workers and those cases recorded by clinics as newborns and infants who would be brought in for medical care. This data was only available for three hospitals and eight clinic areas. Of note, referrals and self-initiated hospital births were recorded by the hospital, but were recorded as being from the mother’s clinic’s service area, suggesting that a few births may have been recorded twice.

Calculations (2X2 comparisons using a Chi-squared test and confidence intervals) were undertaken using Epi Info 7.0. Design effects (DE) associated with the cluster sampling in the baseline survey were estimated using WinPepi Version 11.65. Rates were based on people living in the home at the time of the interview for the surveys and average population for the monthly monitored cohort.

The monitored surveillance population varied over time. The denominator used in rate calculations was the monthly average during 2019. Initially, the household composition from baseline survey was used to define the denominator of the cohort, and was adjusted based on births, deaths and migration reported. Formal household composition interviews were repeated after 8 and 15 months of monitoring.

Ethical review was undertaken and received by Columbia University. Local permission was solicited and received from the Prefecture Ministry of Health Office, and the chiefs of each village before the work began. This study was funded by the US Centers for Disease Control and Prevention.

## Results

In the areas where IRC worked, 29 clusters of 15 households were interviewed and enrolled in the monthly surveillance program. In all 30 areas, 15 additional households were interviewed in the baseline survey. One house refused to be interviewed. For the 11 sites where the team was able to monitor empty houses for the entire areas, about ½ of the houses were not home (Average 52.7%, Median 40.0%, range 21.1 to 70.0%). Not at home may have meant empty that day, but most were believed to be abandoned, or empty for months at a time. For the sake of efficiency, investigators made only minimal efforts to explore the house status with neighbors. Those 896 interviewed households included in the baseline survey contained 4243 people (4.7 people per household); 2244 were females (52.9%) and 983 (23.2%) were reportedly less than 5 years of age.

One hundred fifty-three births were reported in these baseline survey households over the preceding 7.7 months. Reportedly, only 5 of these births (3.3, 95% CI: 1.6–5.0%) occurred at home. This equates to 5.6% of population being born per year (95% CI:4.5–6.7, DE = 1.54) or just under ~ 5.4% of the population (the difference because of two sets of twins and a stillbirth, and a slightly changing denominator) giving birth per year. This survey likely under-recorded stillbirths.

One hundred thirty-six deaths were reported in the baseline survey households over the preceding 7.7 months, which equates to 4.2 deaths / 1000 / month (95%CI: 3.3–5.0, DE = 1.67) or 50.4 deaths / 1000 / year. Thirty-seven deaths were reported over the past 7.7 months among family members < 5 years of age which equates to a rate of 5.0 deaths / 1000 / month (95% CI: 2.5–7.5) or 60 deaths / 1000 / year. The cause of death as reported by the family are shown in the figure below.

The 240 “matched control village” households interviewed as part of the baseline survey had 1190 residents, 617 (51.8%) were females, and 264 (22.2%) were children < 5 years of age. Those households had experienced 48 births (6.3% annual birth rate, 95% CI: 3.8 to 8.9%,), 35 deaths (3.8 deaths / 1000 / month, 95% CI: 1.2–6.5) over the preceding 7.7 months, and 14 of the 48 births (29.2, 95% CI: 20.3 to 38.1%) reportedly occurred at home.

Over the 17 months from October 2018 until February 2020, 435 of the enrolled surveillance households were visited on a monthly basis (because of hostilities, one cluster was dropped) by the same monitor each month, who was in most cases, an individual from their community. Over the course of monitoring, approximately 26 households moved away and on average monthly data was collected for 1804 people. These households reported 127 births (5.0% per year), 41 of which (32.3, 95% CI: 26.3–38.2%) reportedly occurred at home.

As shown in Table 1 below, in the 8 villages surveyed during the baseline visits, where IRC did not work, interviewees were 12 times more likely to report a home birth over the exact same period. This suggests a problem, not with the general reporting of demographic events (the death rates between the served and control areas were similar as were the death rates in the baseline and surveillance data) but specifically with households reporting if a birth was attended.

**Table 1:** Attended and home births in areas served by IRC vs. control areas

	Attended birth	Home birth
IRC area	148	5
Control area	34	14

OR = 12.2 (95% CI: 3.7–32.9) χ^2^ = 28.6.

Table 2: Births reported in baseline survey in Served vs. Control areas, same recall period.

**Table 2:** Births reported in baseline survey of the Served population vs. reported in monthly surveillance during the following year

	Attended birth	Home birth
IRC area Baseline	148	5
IRC area Surveillance	86	41

OR = 14.1 (95% CI: 5.3–37.1) χ^2^ = 46.6.

While not the same time period (essentially 2018 vs. 2019), Table 2 shows that the same households were 14 times more likely to have a home birth recorded by ongoing monthly monitoring compared to an initial baseline survey. These differences in Tables 1 and 2 are extremely significant (*p* < .00001 based on χ^2^).

The difference between the baseline survey and surveillance system with regard to the fraction of births occurring at home (3% vs. 32%) was rather striking. This triggered an assessment of IRC’s administrative data reported by clinics, hospitals and health outreach workers.

Between October 2018 and July 2019, 3 hospitals and 8 clinics with birthing capacity reported 2085 attended births and 109 (5% of total) births that were known to have taken place at home. These 11 facilities served an estimated population of 93,754. This suggests a total annual birth rate of 2.8% and an attended birth rate of 2.67% per year of the total population. While data was not available for some months in 2019, the reports from all of 2018 were similar for the 11 facilities (2299 attended births, 226 (9% of total) home births recorded, in a population 94,000, for a birth rate of approximately 2.8%).

Table 3 below highlights the birth rates and the fraction of births occurring at home in the baseline survey where IRC worked, in the matched control villages where no NGO supported the clinic, and from the administrative sources available.

**Table 3:** Summary of sources of data regarding births in Ouham-Pende and reported results

Source	Births/denominator	Annualized Birth rate %/yr.	% births at home	Reported pop. Represented
8/18 baseline survey of IRC service pop.	153 births / 4243 sample	5.6%	3%	93,754
8/18 baseline survey of 8 non-served matched control villages	48 births / 1190 sample	6.3%	29%	~ 16,000
Community surveillance data IRC service pop. Oct. 2018 – Feb. 2019.-.	127 births / est. 1804 sample pop. on average	5.0%	32%	93,754
IRC clinic based administrative estimates Oct. 2018 – July 2019	2194 births/ 93,754 population served	2.8%	5%	93,754

The National Ministry and Health and WHO and others estimate that births occur at an annual rate of 3.5% of the population [10].

## Discussion

Household surveys are a standard method for assessing health conditions in humanitarian settings, with the SMART method being especially widespread (www.smartsurvey.co.uk/). Various triangulation efforts have found them likely as effective at recording demographic events as more labor-intensive list construction or exhaustive explorations [11]. Having local people visit houses on a regular basis to record demographic events has been shown in this region to be more sensitive and specific than surveys in recording births and deaths [9]. In this case, a household survey produced a birth rate very close to that recorded by a prospective community surveillance process. What was most contrasting between the survey and the community surveillance data was the fraction of births that reportedly occurred in a health facility and thus were attended by an official health worker (See Table 3 above).

In this case, it is almost certain that the monthly surveillance estimate recording more than one third of births were not attended is the more correct estimate. This is said for two reasons; the evidence of data completeness implied by the birth rates and the higher quality of the monthly surveillance encounters.

Reportedly, 23.2% of the population in the baseline survey was less than 5 years of age. This suggests a baseline birth rate between 5 and 6%, not the 3.5% birth rate used as the default assumption by the government, and that permitted program officials on the Prefecture level to assume that the administrative data was complete. The IRC administrative estimate of attended births for these areas corresponds to an implausibly low annual birth rate of 2.8%, close to the 3.5% assumed birth rate used by the government.

While the Government assumes that the birth rate is 3.5% per year, this estimate is unquestionably very low. For example, a nationwide SMART Survey conducted by UNICEF in Dec. of 2019 estimated that 21.7% of the total population was < 5 years [12]. An unpublished Nationwide SMART survey in 2018 put the fraction at 24.7% and our baseline survey in Ouham-Pende put the fraction at 23.2%. Note, that if the annual birth rate was indeed 3.5% of the population, if there were never any infant and child death the fraction of the population that would be children < 5 years would be 17.5% or perhaps slightly higher if older people died off. While surveillance has widely been documented to be incomplete in conflict settings, a 40% nationwide underestimate of births has profound implications for estimates of birth attendance and vaccination coverage. Most importantly for this analysis, the fraction alive under 5 and the surveillance recorded birth rate suggests that many births, potentially half of births, go unrecorded by the administrative data of the health system in Ouham-Pende.

Separately, the surveillance was conducted by someone familiar with the family and village, who was building up rapport from ongoing visits, while the baseline survey interview was with a stranger at the time. It makes sense that reporting would be more complete when reporting to a neighbor.

The question arises, why do people under-report home births when speaking with interviewers in a survey just in the IRC served areas? While the answer is not certain, it is likely that households over report expected behaviors. This has been documented, for example with handwashing in Ethiopia [13]. In the case of our baseline survey, the interviewers arrived on IRC motorcycles, were working with IRC, and everyone knew that IRC was an ardent supporter of in-facility birthing. In fact, IRC had built maternal facilities in five of these communities with great fanfare, they were willing to pay all costs for maternity related care, and their health outreach workers actively shared safe birthing messages. This under-reporting of home births, by a factor of 12, should serve as a cautionary tale for NGO’s in such settings that conduct surveys on topics for which they are known to advocate. The fact that this level of under-reporting did not happen in the comparison villages surveys where no NGO provided health services suggests this under-reporting was, at least in part, induced by the presence or actions of the NGO in the areas they serve. Further research is needed to understand how much of this deception is meant to be gratitude and encouragement to the health providers versus how much is fear or stigma related. Moreover, comparisons of surveys done by NGO’s providing services in very poor areas should be made with interviews done by impartial un-involved interviewers (like a local University) to understand the extent to which beneficiaries deceive interviewers to allow them to hear what they want.

In this scenario with three different insights into the fraction of births that went unattended, no one source was implausible when seen alone. The administrative data suggested an annual birth rate of 2.8% with few births occurring at home. Because the Government and UN claim a birth rate of only 3.5%, this data alone seemed plausible. The survey data within the IRC service area seemed to confirm that few births were unattended, but found a birth rate twice as high as the administrative data. Thus, the baseline survey data estimating a birth rate of 5.6%, with 3% of those births occurred at home, in the absence of the other two sources, seemed plausible. The surveillance data found a birth rate similar to the baseline survey, but found more than 14 times the fraction of births occurring at home compared to the interviews in the baseline survey. This implies two problems, administrative surveillance data are missing a large fraction of all births, and household surveys within the IRC served areas resulted in people not admitting that births occurred at home when they clearly did. The tendency for surveillance to be very incomplete in such settings has been documented as has the tendency of interviewees in surveys to report what is desired or expected of them [8, 13–15] The contrasts between these three birth event measures (a baseline survey, community-based monitoring, and administrative data), highlight the need to compare survey data with other sources (e.g. midwife records in a village of known population, expected birth rates, administrative estimates, official surveillance data) to make sure that major inaccuracies are not arising in the data.

### Limitations

There are many limitations to all aspects of this assessment. The villages and towns where IRC worked were not random and were largely selected by the Ministry of Health. The baseline survey covered a 7.7 month period before the period of household surveillance and there is a small chance that changes in birthing-related habits occurred over the study period, although this does not explain the Served vs. Control area contrasts in the baseline survey. It was the initial study design that the baseline survey would be repeated at the end of the surveillance process so that another survey vs. surveillance comparison could be made that was covering the exact same period. But, the arrival of COVID and associated restrictions and increased violence has prevented that from occurring. The surveillance process has been continued an additional year and a final household survey will be conducted when conditions allow.

The fact that half of households were abandoned or not at home on the day of the baseline interviews may allow for sampling bias. While this would not affect the comparisons between baseline surveys, but could potentially create a difference between baseline survey and surveillance rates. The fact that on average, the surveillance monitoring clusters of 15 houses tended to have approximately four occupied interspersed households that had been missed in the baseline recruitment suggests most of the empty houses were abandoned. It is possible in the community-based surveillance data that the monthly visitations have changed household behaviors as they sense they are being monitored or judged. This seemed to be occurring with increased hospital births in monitored households in a previous similar project.^9^ Some patterns of under-reporting by households in the monthly surveillance have been detected. For example, some households seem to have intentionally not reported pregnancy loss and neo-natal deaths. The surveillance system lost approximately 10% of households over the first 2 years of this project and these households are believed to have disproportionately experienced deaths that triggered the departures, often violent deaths. Anecdotal observations by the monitors suggest it is possible pregnant women are also more likely to move away from this particularly violent area. The effect of this loss-related bias on birth rates of home birthing is unknown. Finally, the administrative data reported to IRC by clinics and hospitals may be incomplete, or lost in transfer. This could artificially reducing the administrative data implied attended birth rates, making the difference between that data and the monthly surveillance data arising because of better data management in the monthly surveillance process rather than intentional under-reporting by households or health workers.

## Conclusion

This comparison of a baseline survey with community-based surveillance and administrative surveillance suggests that households in the Central African Republic grossly under-report births that occur in the home in an area where in-facility birthing was actively promoted by an international NGO that supported the health system. It is believed that interviews in the baseline interview with a stranger induced interviewees to say what they thought the interviewers wanted to hear. Community-based surveillance appears to have advantages in acquiring information about behaviors like attended births.

## Data Availability

The baseline survey dataset for the current study are available from the corresponding author on reasonable request.

## Appendix 1

Questionnaire & Guide.

CAR Community Pregnancy, Birth, Death and Migration monitoring project.

(*just read italicized questions to interviewee)*

***Bold are instructions for interviewer***

1. **Make sure you read the consent script & get consent before starting. Record house number, interview date.**
2. *Starting with the youngest, could you please tell me the age and sex of all the people who are in your household? When I say household, I mean slept here under the same roof with you the last two weeks.* **If in doubt, record any details and discuss with supervisor later. Record age and sex of all and confirm total number with interviewee at end.**
3. *Have any of the members of this household been pregnant since this past Christmas (2017)?* **Record the total number and details (name and age of the women, still ongoing)**
4. *Have any of the members of this household given birth since this past Christmas (2017)?* **Record the total number (twins count as two) and details (who was the mother, place of delivery)**
5. *Have any of the persons in this household died since this past Christmas?* **If yes, record the age and sex of the deceased, the date of death (or you can use the approximate month if people do not know not the exact date), and the cause of death. Record this for all deaths if more than one. Record the location of the death if it was before their arrival.**
  **If a death occurred, did they go to a medical facility for this illness during the month before they died?**
6. *The last time someone in your house was so sick that they could not work or go to school, did they go to a medical facility? If so, which one. How long ago was this?* **Write the details of the illness and if they went somewhere, where and when they went.**

**Thank them for their time and for those households entering the surveillance process, confirm that it would be OK to return once a month and see how the family is and if anyone has come or gone.**

## Abbreviations

CAR: Central African Republic
CI: Confidence Interval
IRC: International Rescue Committee
NGO’s: Non Governmental Organizations
UNICEF: United Nations International Children’s Emergency Fund
WHO: World Health Organization
